# Neighborhood effects on the health of elderly persons: evidence from China

**DOI:** 10.1186/s12877-023-04609-3

**Published:** 2023-12-21

**Authors:** Xia Dongping, Gong Rengui, Hu Yangming, Hu Zan, Xiang Hua

**Affiliations:** 1 College of Public Administration and Law, Hunan Agricultural University, Changsha, China; 2 School of Public Management and Law, Anhui University of Technology, Ma’anshan, China; 3 School of Public Administration, Hunan Normal University, Changsha, China; 4 Hengyang Medical School, The Affiliated Changsha Central Hospital, University of South China, Changsha, China; 5 Hengyang Medical School, Maternal and Child Health Hospital of Changsha County, Changsha, China

**Keywords:** Neighborhood effects, Physical health, Mental health, Elderly persons, Community environment

## Abstract

**Background:**

Presently, global aging has become increasingly serious, whereas the health concerns brought by aging have become a public issue that warrants an urgent solution from all countries across the world. Therefore, this research paper discusses the influence of neighborhood health on elderly individuals’ health, and extending a realistic basis for the other economies to improve the neighborhood environment and promote the health of the elderly.

**Methods:**

Based on the data of CHARLS2018, this research paper adopts the samples that fulfill the study requirements (N = 7326). we constructed a comprehensive research framework integrating oprobit regression model, heterogeneity analysis, conditional mixed process(CMP)robustness testing, Furthermore, the KHB decomposition method is implemented to ascertain the influential mechanism of NMH and NPH on the mental- and physical health of elderly persons.

**Results:**

The oprobit regression model analysis indicates that NMH 0.434 and NPH 0.550 exert positive influences on the elderly’s mental- and physical health. Meanwhile, the effects of conditional mixed process on NMH and NPH stand at 0.381 and4.372, which are different from the oprobit regression results; thereby, indicating the existence of endogeneity. Afterward, KHB mediating effect confirms that Internet use, gift reciprocity, and charity activity contribute 30.21% and 16.83% to mental- and physical health, respectively.

**Conclusions:**

Firstly, the NMH and NPH demonstrate a positive influence on the mental- and physical health of the elder population. However, there exist heterogeneous differences. Secondly, the conditional mixed process deals with the endogeneity of NMH and NPH. Thirdly, social integration, social interaction, and social engagement serve as significant transmission mechanisms for the influences of NMH and NPH on the health of elderly persons.

## Introduction

China’s National Health Commission predicts that by 2035, 30% of China’s population will be over 60 years old [1], China is facing a serious problem of aging population. The health issues of the elders are not only associated with their well-being but also with the stability of the national health system. Therefore, in recent years, the health of the older generation has become a hot topic in academic circles.

The previous studies focus on the influence of individual factors on mental and physical health, such as gender, education, income, occupation, IDA, and other factors [2, 3]. As a result of this, there is a continuous discussion that asserts a need to take into account not only individual characteristics but also attributes of the background and group to which an individual is related, in order to understand the health and disease distributions [4, 5]. Therefore, the neighborhood effect has been able to attract wide-scale attention. Meanwhile, the neighborhood effect theory refers to the impact of different persons residing together, on the people in the neighborhood effect [6]. For instance, significant concentrations of poor persons further lead to neighborhood degradation, lack of infrastructure, educational inequality, and stigma [7–9]. whereas the health of individuals is seriously influenced who reside in such adverse neighborhoods for a long period [10]. Furthermore, certain scholars have also proposed that the social- and physical environment of neighborhood demonstrate a significant effect on the chronic diseases and mental ailments [11, 12]. In further studies, researchers point out that residents’ subjective perception of the neighborhood environment exhibits a substantial influence on the happiness and health status of residents [13, 14]. Neighborhood interaction can meet their social needs and enhance their sense of belonging [15, 16]. This helps reduce loneliness and social anxiety in older adults, which positively affects their mental health [17, 18]. The research scholars also attempt to analyze the regulation mechanism of neighborhood influence on human health. For instance, neighborhood social participation presents a critical mechanism of neighborhood influence on health [19, 20]. Meanwhile, the mediating effect of neighborhood social interaction exhibits an influence on the neighborhood’s economic status and mental health [21]. Similarly, there is also a significant mediating role of social capital in neighborhood environmental perception and depression [22]. Besides this, the community-building environment demonstrates a positive impact on the health of elderly people through outdoor exercise [23].

Although researchers have achieved more results on neighborhood effects. However, there are some shortcomings in these existing studies, such as more bias towards the impact of the neighborhood built environment on health, and much weaker research on the impact of the neighborhood social environment on health. Moreover, the neighborhood effect is not well studied for the elderly population as a vulnerable group. Therefore, this paper selects CHARLS2018 data to analyse the relationship between neighbourhood health and the health of elderly individuals by using the oprobit model, to validate the mechanism role of social integration, social engagement and social interaction in the process of neighbourhood health affecting the health of the elderly and to test the heterogeneity of the samples of different groups.

## Method

### Design

This is a “selective relevance” design and summarises the design. The China Health and Retirement Longitudinal Study (CHARLS) 2018 was organised by Peking University to investigate [24], and the survey was conducted among older Chinese people (aged 45 and over) families and individuals. The purpose of the study is to use the collected data to analyze the upcoming aging issues in China and to enhance interdisciplinary research on aging. The baseline survey was conducted in 2011 and has been tracked every two to three years since. The study area is 150 county-level units, 450 village-level units, and 17,000 people in about 10,000 households in China. The project used multi-stage sampling, with PPS sampling methods at both the county/district and village sampling stages. CHARLS pioneered the electronic mapping software (CHALRS-GIS) technology to produce village sampling frames using the map method. The content design of the questionnaire is also consistent with the contents of the aging survey series in countries around the world, and considering the important position of community elderly care in China’s elderly care model, the questionnaire especially adds the part of community facilities and services for the elderly.

All CHARLS investigations have received ethical approval from the Institutional Review Committee of Peking University. The IRB approval number for the Main Household Survey (including anthropometry) is IRB00001052-11015; The IRB approval number for the biomarker collection is IRB00001052-11014.

### Sample

In accordance with the research needs, data from older individuals (aged 60 and above) are chosen while the outlier samples and key missing information are removed from this study. Finally, mixed 7,326 cross-section mixed-samples of older persons from 126 cities (prefecture-level) are adopted in this paper. Of these, 3,764 were female, and 3,562 were male. The mean age was 68.213 years.

### Variables

#### Explained variable

The mental- and physical health of elderly persons are explained variables in this research paper. Mental health is estimated with the help of a simplified CES-D Depression scale in the CHARLS questionnaire. For example, if you worry about small things, concentration, low mood, sleep, loneliness and so on. Finally, mental health scores are measured based on the respondents’ responses.

In the CHARLS questionnaire, by asking the participants certain questions related to the functional limitations and helpers. Whether there are difficulties with these behaviors, jogging and walking, standing up from a chair while sitting for a long time, climbing stairs continuously etc., these questions were used to evaluate the physical health of the elderly.

#### Explanatory variables

NMH and NPH are adopted as core independent variables in this paper. This paper draws on the reference to the previous research [25, 26],The community neighborhood mental health is defined as the average mental health level of other elderly people in a community except the elderly themselves. The definition of NPH is the same as NMH.

#### Mediating variables

Social integration, social interaction, and social engagement are the mediating variables of this study. Owing to the continuous advancements in the Internet era, Internet use has become an imperative tool for older individuals to integrate into society [27]. For this purpose, Internet use in the previous month is adopted as a social integration’s proxy variable. In addition to this, the participation of older individuals in charity practices also represents a parameter to gauge the social participation of elderly citizens while participation in charity practices is employed as a proxy variable for social engagement. Since reciprocation of gifts is a customary social culture in China, therefore, gift reciprocity over the previous year is taken as a social interaction proxy [28].

#### Control variables

In this paper, the influence of personal characteristics, family factors, and public medical policy is excluded in order to effectively study the neighborhood effect [29, 30]. Consequently, 13 variables including age, gender, education, habitation, marital status, ln(total personal income), ADL, IADL, percentage of adult sons, accompanying time from adult children, economic support from adult children, medical service satisfaction, and family doctor services are incorporated as control variables in this study.

### Procedure

CHARLS begins with stratified and four-stage (county/district-village/community-household-individual) random sampling by GDP per capita in urban districts and rural counties. Next, all sampled households were screened to ensure that all sampled households had age-eligible members. If there are eligible members in the sampled households then a random selection of family members can be interviewed. If the person being interviewed is 45 years of age and older, then the person being interviewed becomes the primary respondent and their spouse is interviewed. In this paper, when using CHARLS2018 data, elderly people living in the same community aged 60 years or older were selected and the sample was screened to meet the requirements through some Functional Limitations and Helpers questions in the CHARLS questionnaire.

### Statistical analysis

In this paper, oprobit regression model was used to investigate the relationship between neighborhood health and elderly health. In addition, 95% confidence intervals (CL) are reported. The endogeneity test of Neighborhood Sport and Neighborhood Entertainment Activities was analyzed using conditional mixed process. The robustness test of oprobit model was performed by using the ologist model. Based on the KHB model, the effects of social integration, social engagement and social interaction as mediating variables on the physical and mental health of elderly individuals were analyzed. All analyses were performed using the stata17 version. Statistical significance was defined as P < 0.05.

## Results

### Descriptive statistics

Table 1 populates a descriptive statistical analysis of study variables; thereby, displaying the distribution information of each variable. In this paper, regression analysis is carried out on 7326 sample sizes.

**Table 1:** Descriptive Statistics

Variable	Number	Percentage	Variable	Number	Percentage
**Gender**			Medical Service Satisfaction		
Male	3562	48.62	1	650	8.87
Female	3764	51.38	2	563	7.68
**Marital Status**			3	3077	42
With Spouse	1518	20.72	4	1755	23.96
Otherwise	5808	79.28	5	1281	17.49
**Educational Achievement**			**MH**		
0	3614	49.33	1	195	2.66
6	1701	23.22	2	1349	18.41
9	1262	17.23	3	4043	55.19
12	446	6.09	4	1739	23.74
15	303	4.14	**PH**		
Habitation			1	510	6.96
0	2838	38.74	2	2015	27.5
1	4488	61.26	3	4223	57.64
**ADL**			4	578	7.89
1	15	0.2	Variable	Mean	SD
2	83	1.13	NPH	3.419	0.165
3	1536	20.97	NMH	2.719	0.348
4	5692	77.7	NS	0.058	0.060
IADL			NEA	0.147	0.093
1	45	0.61	Internet Use	0.117	0.117
2	433	5.91	Charity Activity	0.058	0.060
3	6402	87.39	Ln (Gift Reciprocity)	3.444	3.660
4	446	6.09	Age	68.213	6.181
**Family Doctor Services**			lnTPI	7.268	2.996
0	6993	95.45	PAS	0.544	0.302
1	333	4.55	lnESC	4.085	3.467
			Accompanying Time	2.147	3.072

### Baseline regression analysis

The NPH exhibits a significantly positive correlation with the physical health of elderly individuals, after controlling the interference of their family factors, basic personal characteristics, and public medical policies, as illustrated in Model [1] Table 2 the result 0.975. Further, once all the control variables are added on the basis of Model [1], the authors find out that the result 0.550 in Model [2] is still significant. Moreover, the same approach is used to confirm whether NMH demonstrates a significant influence. The NMH results of Model [3] are obtained 0.556 when the interference impact is controlled by the researchers. In addition to this, the result 0.434 of Model [4] are derived by regression estimation on the basis of Model [3], thereby, indicating that the NMH exhibits a significantly positive association with the elderlies’ mental health. At the same time, the authors discover that NMH and NPH jointly demonstrate a significantly positive relationship with the elderly’ mental- and physical health. Specifically, in terms of the control variable in Table 2, gender(using female as a reference)male 0.296 and 0.500, marital status (using otherwise as a reference) with spouse 0.184 and0.064, lnTPI 0.016 and 0.030, lnESC 0.013 and 0.005, accompanying time 0.019 and 0.006; the proposed variables demonstrate a significant positive association with elder’s mental and physical health. Contrary to this, habitation (using otherwise as a reference) agricultural 0.133 and 0.078, and age 0.009 and 0.031 records an indirect impact on the mental- and physical health of senior citizens. Although, the proportion of family doctor’s services and adult sons revealed an insignificant impact on their mental and physical status.

**Table 2:** Oprobit regression model

	-1	-2	-3	-4
	PH(one)	PH(full)	MH(one)	MH(full)
**NPH**	0.975^***^	0.550^***^		
	[0.823,1.128]	[0.377,0.724]		
**NMH**			0.556^***^	0.434^***^
			[0.484,0.629]	[0.352,0.515]
**Gender(using female as a reference)**				
Male		0.500^***^		0.296^***^
		[0.441,0.559]		[0.239,0.352]
Age				
		-0.031^***^		-0.009^***^
		[-0.036,-0.026]		[-0.013,-0.004]
**Marital Status(using otherwise as a reference)**				
With Spouse		0.064^*^		0.184^***^
		[-0.007,0.134]		[0.117,0.251]
**Educational Attainment(option below primary school for reference)**				
primary schools		0.075^**^		-0.058
		[0.000,0.150]		[-0.132,0.017]
junior middle school		0.110^***^		0.028
		[0.032,0.188]		[-0.047,0.103]
senior high school		0.184^***^		0.142^***^
		[0.096,0.272]		[0.061,0.224]
University and above		0.332^***^		0.196^***^
		[0.212,0.452]		[0.073,0.320]
**Habitation(using otherwise as a reference)**				
Agricultural		-0.078^***^		-0.133^***^
		[-0.138,-0.019]		[-0.193,-0.072]
lnTPI				
		0.030^***^		0.016^***^
		[0.021,0.040]		[0.007,0.025]
**ADL(using unable to complete as a reference)**				
No difficultiy		-7.355^***^		-0.789^**^
		[-7.639,-7.071]		[-1.405,-0.172]
Difficulties but still achievable		-2.154^***^		-0.777^***^
		[-2.438,-1.869]		[-1.054,-0.500]
Difficulty and need help		-1.186^***^		-0.604^***^
		[-1.256,-1.116]		[-0.672,-0.535]
**IADL(using unable to complete as a reference)**				
No difficultiy		-1.251^***^		-0.487^***^
		[-1.653,-0.849]		[-0.850,-0.124]
Difficulties but still achievable		-1.197^***^		-0.550^***^
		[-1.366,-1.027]		[-0.712,-0.388]
Difficulty and need help		-0.126^**^		0.073
		[-0.242,-0.010]		[-0.041,0.186]
PAS				
		0.057		-0.035
		[-0.031,0.145]		[-0.120,0.050]
lnESC				
		0.005		0.013^***^
		[-0.003,0.013]		[0.005,0.020]
Accompanying Time				
		0.006		0.019^***^
		[-0.003,0.015]		[0.010,0.028]
**Family Doctor Services(using otherwise as a reference)**				
Receive the Paid Family Doctor Services		-0.066		-0.036
		[-0.199,0.066]		[-0.169,0.096]
**Medical Service Satisfaction(use very good as a reference)**				
Very Bad		-0.312^***^		-0.485^***^
		[-0.427,-0.197]		[-0.591,-0.378]
Bad		-0.253^***^		-0.296^***^
		[-0.369,-0.137]		[-0.411,-0.181]
Neutral		-0.104^**^		-0.173^***^
		[-0.183,-0.025]		[-0.250,-0.096]
Good		-0.083^*^		-0.090^**^
		[-0.170,0.004]		[-0.176,-0.004]
Pseudo R²	0.01	0.206	0.014	0.087
N	7326	7326	7326	7326

*Note*: The coefficient is OR value (odds ratio). *p < 0.1, ** p < 0.05, *** p < 0.01, 95% confidence interval are in parentheses

### Endogenous analysis

In this study, the chosen instrumental variables are neighborhood sports and neighborhood entertainment activities (both are measured in the same manner as NPH) since as an instrumental variable there is a need to satisfy both the exophytic and dependence conditions. Meanwhile, sports and entertainment practices only impact the health status of the senior citizen who participates in such practices [31]. Nevertheless, there is no such influence on other elderlies who do not participate; thereby, fulfilling the two requirements of instrumental variables. Specifically, Table 3 depicts the estimation results of the conditional mixed process obtained by the instrumental variable technique. The regression results of the first stage suggest that NS stands with 1.214 and 0.164. Parallely, the regression estimations of the first stage reveal that NEA stands with 0.434 and 0.214, Based on this, both NEA and NS represent significant impacts on the NMH and NPH. This also confirms that the NEA and NS fulfill the correlation hypothesis. Simultaneously, different statistical tests are applied to evaluate the instrumental variables. Using CMP estimation, the atanhrho_12 parameter was significant at both NMH 0.112 and NPH-0.721 (passing the 1% significance level test), showing that CMP estimation is more efficient and results are more accurate.

**Table 3:** Conditional mixed process (CMP)

	First-Stage OlS	Second-Stage Oprobit	First-Stage OlS	Second-Stage Oprobit
	NPH	PH	NMH	MH
NPH		4.372**		
		[3.974,5.350]		
NMH				0.381**
				[0.204,0.559]
NS (Neighborhood Sport)	0.164***		1.214***	
	[0.088,0.240]		[1.079,1.349]	
NEA (Neighborhood Entertainment Activities)	0.214***		0.434***	
	[0.154,0.275]		[0.352,0.515]	
Control Variables	YES	YES	YES	YES
atanhrho_12	-0.721***		0.112***	
	[-0.980, -0.462]		[0.084,0.140]	
*N*	7326			

*Note*: Similar to the Note in Table 2

### Robustness test

#### Replacement of the explained/dependent variable

Firstly, mental- and physical health are replaced with life satisfaction, through an oprobit model estimation. The results 0.379 and 0.508 are achieved, which reflects that life satisfaction still reports a significantly positive influence. Secondly, the oprobit model is adopted to replace mental- and physical health with self-rated health. Accordingly, the estimation results confirm that 0.325 and 0.462, which still demonstrates a significantly positive relationship with mental- and physical health/wellbeing. Thirdly, the oprobit model regression is utilized to replace the mental- and physical health with 3-year self-rated health changes; consequently, deriving 0.150 and 0.508. This shows that the 3-year self-rated health changes also exert a significant positive influence.

#### Alternation of the explanatory/independent variable

In the previous stage of this study, NPH and NMH data at the community level are used for regression estimation. Although, a larger range of county-level NMH and NPH is used instead of community-level NMH and NPH. Accordingly, the county-level NMH 2.243 and NPH 2.243 show significantly positive influences on the mental- and physical health/wellbeing of the senior citizens.

#### Test of different samples

In the context of whether elderlies had been hospitalized in the past year, older persons are categorized into two different groups: those individuals who had been hospitalized in the past year (1429 samples) and those individuals who had not been hospitalized in the past year (5897 samples). Meanwhile, the impact of NMH and NPH on the mental- and physical health of senior citizens, is analyzed once again with different size samples. 1429 respondents who had been hospitalized in a year were influenced by the mental- and physical health of their neighbors 0.897 and 1.042. Moreover, the NMH and NPH of 5897 individuals who had not been hospitalized in one year are 0.786 and 1.256.

### Mechanism analysis

The variables including social integration, -engagement, and -interaction are selected as mediating variables in this research. Afterward, the mediation effect test is undertaken by using the KHB method, which is able to decompose the total influence of NMH effect on the mental well-being of elderlies into direct- and mediating impacts, while anticipating the contribution and magnitude of NMH impact on the derived variables by each mediating path. Similarly, Internet use, gift reciprocity, and charity activity are adopted as the proxy indicators of social integration, social interaction, and social engagement respectively. The relevant regression estimations are populated in Table 4. Accordingly, Internet use displays a substantially positive influence on NMH and NPH 0.115 and 0.171. In addition to this, charity activity exerts a significantly positive impact on NMH and NPH 0.042 and 0.048. Further, gift reciprocity shows a significantly positive association with the NMH and NPH, with 0.819 and 0.612.

**Table 4:** Influence of NPH and NMH on Mediation Mechanism Variables

		Internet Use	Charity Activity	Gift Reciprocity
Panel A:PH	NPH	0.171***	0.048***	0.612**
		[0.157,0.186]	[0.040,0.056]	[0.087,1.137]
	Control Variables	YES	YES	YES
	Adj-*R*²	0.308	0.229	0.061
	N	7326	7326	7326
Panel B:MH	NMH	0.115***	0.042***	0.819***
		[0.108,0.122]	[0.038,0.045]	[0.558,1.079]
	Control Variables	YES	YES	YES
	Adj-*R*²	0.351	0.261	0.065
	N	7326	7326	7326

*Note*: Similar to the Note in Table 2

The oprobit regression model advocate that the mediating effects of Internet use, charity activity, and gift reciprocity are separately measured in the model; thereafter, the proposed three variables are added to the model at the same time, in order to compute the total mediating effects. Furthermore, the portion of each mediator variable that is possible to explain separately and the portion that can be enlightened in the whole model (Table 5). The Internet use insertion alone into the mediation-based analysis demonstrates that the contribution of Internet use to mental- and physical health stands at 15.13% and 9.29%, respectively. However, when charity activity is alone put into the mediation model, the proposed contribution to physical- and mental health becomes 11.60% and 3.35% respectively. In addition to this, the contribution of putting gift reciprocity into the model alone stands at 3.48% and 4.19%, respectively. Finally, the contribution of the three factors to mental- and physical health is reported to be 30.21% and 16.83%, respectively.

**Table 5:** Effect Comparison and Decomposition of KHB Techniques

	PH		MH	
	(1)	(2)	(3)	(4)
	Mediating Effect	Degree of Contribution	Mediating Effect	Degree of Contribution
Internet Use	1.188***	9.29%	0.619***	15.13%
	(0.989,1.386)		(0.532,0.706)	
Charity Activity	1.193***	3.35%	0.617***	11.60%
	(0.995,1.392)		(0.530,0.704)	
Ln (gift Reciprocity)	1.184***	4.19%	0.612***	3.48%
	(0.986,1.382)		(0.526,0.699)	
Total		16.83%		30.21%

*Note*: Similar to the Note in Table 2

### Heterogeneity analysis of NPH and NMH

**Fig. 1:**
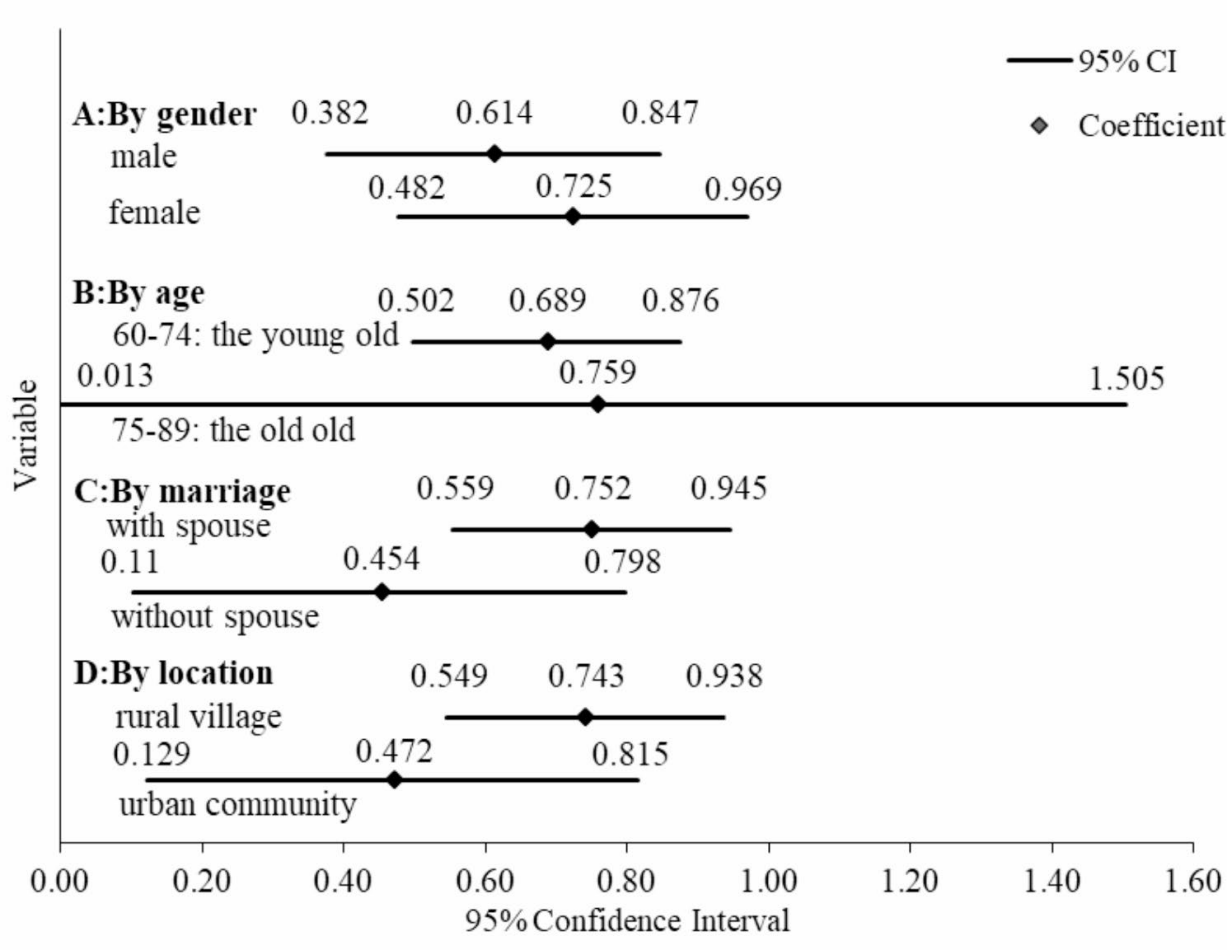
Heterogeneity analysis of NPH

**Fig. 2:**
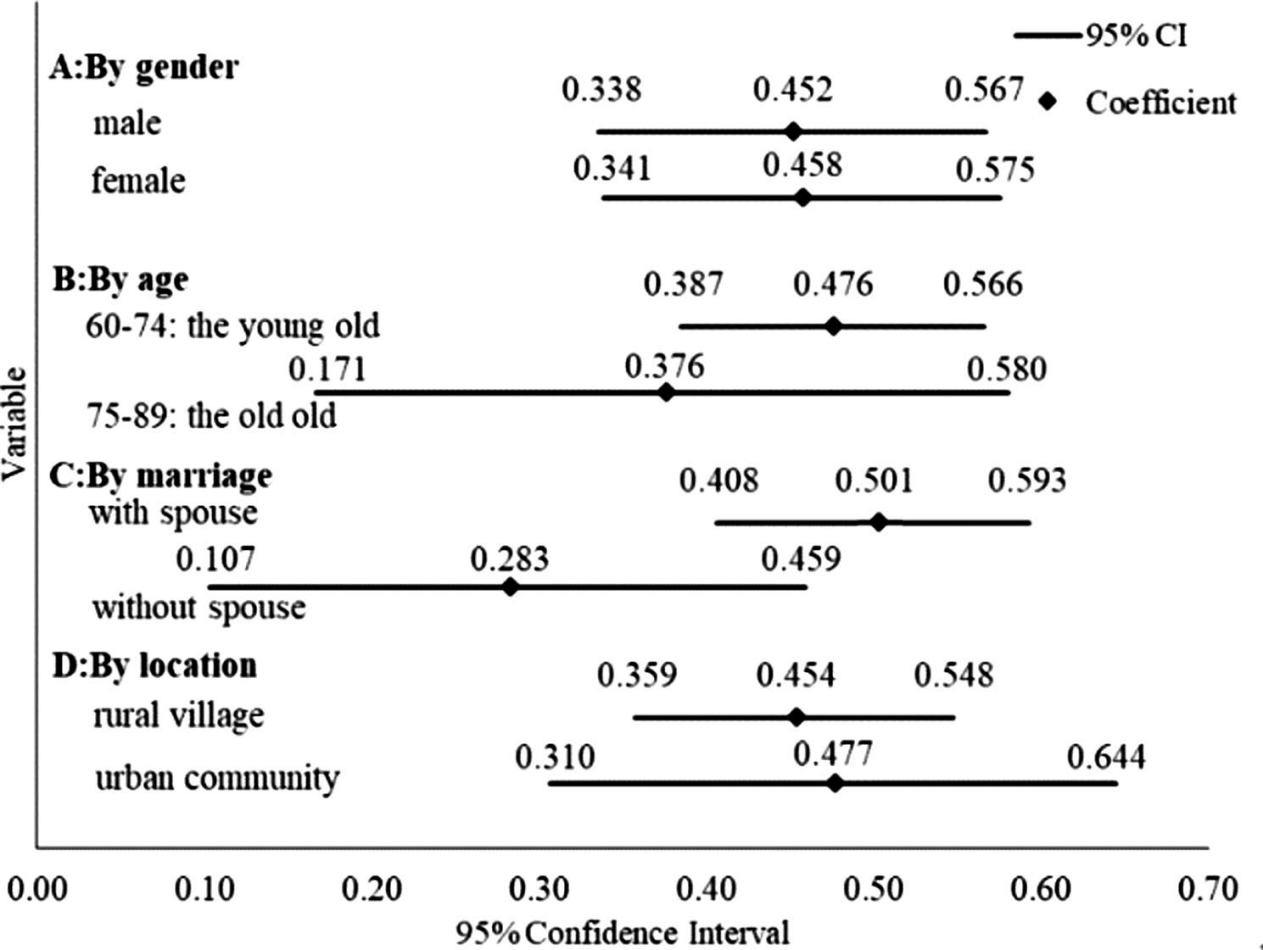
Heterogeneity analysis of NMH

The paper utilizes oprobit regression model to analyze the impact of neighborhood effects on the health of elderly persons by age, gender, marital status, and place of residence to determine whether there is heterogeneity in the impact of neighborhood effects on the health of elderly persons. Subsequently, the results of oprobit regression model results are presented in Figs. 1 and 2. In terms of gender, NMH, and NPH exerts a substantially positive effect on both the female and male populations’ mental- and physical health, while the NMH of the males is recorded to be 0.452 On the one hand, the NMH of females stands at 0.458 which is 0.005 higher than males. On the other hand, the NPH of males is 0.614. Further, the NPH of females stands at 0.725; thus, indicating that females are 0.111 higher than males in terms of NPH. From the perspective of age, the NMH of the young old neighborhood is 0.476 whereas the NMH of the old old stands at 0.376, with the young old 0.100 higher than the old old. Additionally, the NPH of the young old is 0.689 and the NPH of the old old is 0.759; thereby, implying that the old old is 0.070 higher than the young old. In the context of marital status, the NMH of older people with a spouse stands at 0.501. Moreover, the NMH of elderlies without a spouse is recorded to be 0.283. This indicates that the proportion of those with a spouse is 0.218 higher than those without a spouse. Parallel to this, the NPH of elder individuals with a spouse is 0.752whereas the NPH of elder individuals without a spouse is reported to be 0.454. Thus, the number of older people with a spouse is 0.298 higher than those without a spouse. In terms of habitation, on the one end, the NMH of elders in rural villages stands at 0.454; on the other end, the NMH of the elder population in urban communities is recorded to be 0.477. This confirms that the urban area is 0.263 higher than the rural area in terms of the NMH. Finally, the NPH of the elder population in a rural village is 0.743; conversely, the NPH of elders in urban communities is 0.472; thereby, reflecting that the rural area is 0.271 higher than the urban area, in the context of NPH.

## Discussion

### Key finding

oprobit regression model reflects that education, marital status, gender, lnTPI, IADL, lnESC, ADL, medical service satisfaction, and accompanying time demonstrate a significant positive impact on the explained variable. Conversely, regression results reflect a significantly negative association between age and habitation. Meanwhile, the percentage of adult sons and family doctor services show no significant influence on the dependent variable. Specifically, the oprobit regression model of family doctor services are not significant, since the policy of contracting family doctor services is slowly implemented in the whole country. In addition, as per the CHARLS questionnaire, the number of older individuals who participated in the family doctor program account for a small proportion, with 333 respondents included in the study sample. As a result, the effect of the family doctor policy is not captured in this study. Besides this, the insignificance of oprobit regression model results for the percentage of adult sons can be attributed to the fact that a large number of migrant workers entering the city and the rapid urbanization of China has broken the conventional model of raising children for old age [32], therefore the percentage of adult sons exhibits no influence on the elderlies’ mental and physical health.

The influence of NPH and NMH on different elderly groups may be different. Firstly, based on the gender differences, the impact of NMH and NPH on the MH and PH of females 0.458, 0.725 is higher as compared to that for males 0.452, 0.614. This is due to the fact that females score higher than males in relationships [33] and social participation is more active among females [34]. For instance, females experience more collective behavior as compared to those males in group activities such as square dancing, therefore the effect of NMH and NPH among females is stronger. This is consistent with the conclusion proposed by scholar Shan Mao that women have greater cognitive benefits than men in participating in social activities [35]. Secondly, in terms of age, NMH has a greater impact on the young old 0.476 than the old old 0.376, possibly because the young old are more open to trying new things, so NMH has less of an impact on the old old. NPH has a greater impact on the old old 0.759 than the young old 0.689, probably because the young old spend more time on “intergenerational parenting” and less time in group activities than the old old. Thirdly, from the perspective of differences in spouses’ status, the impacts of NMH and NPH on older persons with spouses 0.501, 0.752 function should be greater than the old males of without spouse 0.283, 0.454. Since older adults with spouses socialize not only with familiar people but also with persons that their partners know well; thus, expanding their social network. Based on this, older persons with spouses are more influenced by NMH and NPH [36]. Fourthly, in the context of habitation difference, NMH has a greater impact in urban0.477 than in rural areas0.454, probably because of the greater attention paid to the mental health of older people in urban areas, and the more robust social organisations that regularly organise home visits. NPH has a greater impact in rural 0.743than in urban areas 0.472, probably because the living environment in rural areas is more comfortable and the air and food are healthier than in urban areas.

### Value addition of this study

In order to confirm the robustness of the NPH and NMH effects on the physical and mental health of elderlies, this research study adopts NS and NEA as instrumental variables to ascertain whether NMH and NPH demonstrate an intrinsic impact on elderly person’s mental- and physical health. The endogeneity test parameter atanhrho_12 of the CPM model is significant at the 1% level, which also indicates that the estimate obtained by CMP method is more accurate. This also reflects that NMH and NPH are endogenous to the elderly’ mental- and physical health; thereby, exerting a biased effect on the study outcomes. Besides this, the application of instrumental variables substantially improves the results of baseline regression, while also verifying that the NMH and NPH display a positive impact on the elder generation’s mental health. In addition to addressing the endogeneity problem, the county-level NPH and NMH for NPH and NMH (community-level) are substituted through the substitution of the explanatory/independent variables. The possible reason behind this is that county-level administrative divisions have been practiced since ancient times in China. Further, residents in the same county represent the same regional culture, public policies, and ethnic characteristics. Therefore, the county-level neighborhood health impact is associated with the community-level neighborhood health impact. Resultantly, this paper confirms that the higher level of NMH and NPH also exerts a positive and significant effect on senior persons’ mental and physical health. In addition to this, the dependent variable method is replaced to verify robustness. Consequently, self-rated health, life satisfaction, and three-year health changes are adopted to replace the explained/dependent variables. Firstly, since life satisfaction reflects a positive association with individuals’ physical and mental health [37], therefore, this article attempts to replace mental and physical health with life satisfaction. Secondly, self-rated health highlights the residents’ judgment of their own health, which is a comprehensive and high evaluation method of mental and physical health, therefore this variable can be used as an alternative variable of the dependent/explained variable [38]. Thirdly, the use of three-year health change as the replacement variable is based on the reason that the data used in this research article is cross-section data, therefore a lag effect of historical health is expected. On this basis, the use of a three-year health change can alleviate the aforementioned problem. Consequently, the oprobit model is applied to the regression of the above three substitution variables, whereas the results show that all of them exhibit significantly positive effects. Finally, the authors analyze the robustness of the size sample in this paper, while taking the hospitalization status of older individuals in a year, as a classification standard. Additionally, elderly persons are divided into a small sample hospitalized in a year and a large sample not hospitalized in a year. Reportedly, the results of this study highlight that both large and small samples report significant positive effects.

### Strengths

This paper incorporates social integration, social interaction, and social engagement as mediating variables to further analyze the influence mechanism of neighborhood effects on the physical and mental health of the elderly. Similarly, Internet use, gift reciprocity, and charity activity are taken as the proxy variables of social integration, social interaction, and social engagement, respectively. The study findings indicate that these three proposed variables show significant impacts on the elderly’ mental and physical health. This may be due to the fact that higher social integration, social interaction, and social engagement make older persons feel more social and secure; thus, reducing loneliness, helplessness, and other adverse emotions, while showing more willingness to move outdoors.

### Limitations

There are two notable limitations in this study. Firstly, there is a slight lag in the data. On one hand, the data for CHARLS2018 was collected in 2017, prior to the emergence of TikTok, Temu, and similar apps having a significant impact on the health and socialization of the elderly. On the other hand, the effect of COVID-19 on the physical and mental health of the elderly from 2020 to 2022 has not yet been measured. Secondly, the mediating variables have their limitations. Since the CHARLS survey does not address neighborhood relationships, this study uses gift reciprocity as a proxy for social interaction. Consequently, further research may be conducted to examine the mediating link between the neighborhood effects and the health of the elderly in the future.

## Conclusions

Based on the 2018 data from the China Health and Retirement Tracking Survey (CHARLS), this paper analyzes the influence of neighborhood effects on the health of elderlies. Resultantly, this study not only extends a practical foundation for other economies to improve neighborhood environment and elderly health but also supports the development of an age-friendly country. The study results point out that the elderlies’ mental and physical health shall increase by 0.381 and 4.372 units, respectively, when the NMH and NPH increase by 1 unit. Moreover, social integration, social interaction, and social engagement serve as critical mechanisms for NMH to improve the older generation’s mental and physical well-being/health. In this research article, the heterogeneity of NMH and NPH is further examined; reportedly, the impact of NPH and NMH on females is significantly higher than that on males; further, NMH has a greater impact on the young old than the old old, NPH has a greater impact on the old old than the young old, the effect of NPH and NMH is significantly higher for with spouse elderly persons than for without spouse elderly; finally, NMH has a greater impact in urban than in rural areas. NPH has a greater impact in rural than in urban areas.

Consistent with the conclusions of this research article, the authors propose the following suggestions: Firstly, there is a need to improve venues for community activities. For instance, the community offers comfortable activity venues for older people, which is convenient to organize group activities and fitness exercises; thus, increasing their willingness to perform activities, and eventually promoting their physical and mental health. Secondly, there is also a dire to build smart communities. The construction of a smart community allows the elderly to yield the dividends of science and technology, reduces the anxiety caused by digital trenches, helps older persons to understand the functions and use of smart pension products, and also expands the range of social interaction. As a result of this, older persons would demonstrate a stronger sense of social integration. Thirdly, different measures should be taken to strengthen the construction of community cohesion. Since strong community cohesion promotes the atmosphere of friendship and mutual help between neighbors, in order to enhance the interaction and communication between neighbors, improve the elder individuals’ sense of security, and boost their mental and physical health and well-being.

## Data Availability

The data that support the findings of this study are available from Peking University, but restrictions apply to the availability of these data, which were used under license for the current study, and so are not publicly available, but can be obtained from corresponding author upon request with permission. If researchers want to get the data, you can login China Health and Retirement Longitudinal Study website (http://charls.pku.edu.cn/index.htm) for download.

## Abbreviations

PH: Physical health
MH: Mental health
NPH: Neighborhood physical health
NMH: Neighborhood mental health
NS: Neighborhood sport
NEA: Neighborhood entertainment activities
lnTPI: Ln(Total personal income)
ADL: Activities of daily living
IADL: Instrumental activity of daily living
PAS: Percentage of adult sons
lnESC: Ln(Economic support from adult children)
